# Health-Related Physical Fitness in Healthy Untrained Men: Effects on VO_2_max, Jump Performance and Flexibility of Soccer and Moderate-Intensity Continuous Running

**DOI:** 10.1371/journal.pone.0135319

**Published:** 2015-08-25

**Authors:** Zoran Milanović, Saša Pantelić, Goran Sporiš, Magni Mohr, Peter Krustrup

**Affiliations:** 1 Faculty of Sport and Physical Education, University of Niš, Niš, Serbia; 2 Faculty of Kinesiology, University of Zagreb, Zagreb, Croatia; 3 Sport and Health Sciences, College of Life and Environmental Sciences, University of Exeter, Exeter, United Kingdom; 4 Center of Health and Human Performance, Department of Food and Nutrition, and Sport Science, University of Gothenburg, Gothenburg, Sweden; 5 Faculty of Natural and Health Sciences, University of the Faroe Islands, Tórshavn, Faroe Islands; 6 Department of Nutrition, Exercise and Sports, Copenhagen Centre for Team Sport and Health, University of Copenhagen, Copenhagen, Denmark; University of the Balearic Islands, SPAIN

## Abstract

The purpose of this study was to determine the effects of recreational soccer (SOC) compared to moderate-intensity continuous running (RUN) on all health-related physical fitness components in healthy untrained men. Sixty-nine participants were recruited and randomly assigned to one of three groups, of which sixty-four completed the study: a soccer training group (SOC; n = 20, 34±4 (means±SD) years, 78.1±8.3 kg, 179±4 cm); a running group (RUN; n = 21, 32±4 years, 78.0±5.5 kg, 179±7 cm); or a passive control group (CON; n = 23, 30±3 years, 76.6±12.0 kg, 178±8 cm). The training intervention lasted 12 weeks and consisted of three 60-min sessions per week. All participants were tested for each of the following physical fitness components: maximal aerobic power, minute ventilation, maximal heart rate, squat jump (SJ), countermovement jump with arm swing (CMJ), sit-and-reach flexibility, and body composition. Over the 12 weeks, VO_2_max relative to body weight increased more (p<0.05) in SOC (24.2%, ES = 1.20) and RUN (21.5%, ES = 1.17) than in CON (-5.0%, ES = -0.24), partly due to large changes in body mass (-5.9, -5.7 and +2.6 kg, p<0.05 for SOC, RUN and CON, respectively). Over the 12 weeks, SJ and CMJ performance increased more (p<0.05) in SOC (14.8 and 12.1%, ES = 1.08 and 0.81) than in RUN (3.3 and 3.0%, ES = 0.23 and 0.19) and CON (0.3 and 0.2%), while flexibility also increased more (p<0.05) in SOC (94%, ES = 0.97) than in RUN and CON (0–2%). In conclusion, untrained men displayed marked improvements in maximal aerobic power after 12 weeks of soccer training and moderate-intensity running, partly due to large decreases in body mass. Additionally soccer training induced pronounced positive effects on jump performance and flexibility, making soccer an effective broad-spectrum fitness training intervention.

## Introduction

Health-related physical fitness has been defined as specific components of physical fitness related to body composition, cardiorespiratory fitness, muscular fitness, flexibility and body composition [1], whereas performance related fitness is defined in relation to crucial abilities for success in sports competitions and athletic events [2]. It is well known that aging causes deterioration of all health components if regular physical activity and an active lifestyle are not maintained during the lifespan. In addition, improving or maintaining physical fitness reduces the risk of all-cause and cardiovascular diseases [3]. Despite the fact that fitness is a strong mortality predictor, many studies fail to take into account overall fitness status as an independent risk factor compared to parameters such as hypertension, diabetes, smoking or obesity [4].

Several studies argue that moderate-intensity continuous exercise training (RUN) is an efficient intervention for improving cardiorespiratory fitness regardless of age, training status or gender [5–9]. In addition, meta-analyses reports suggest high-intensity interval training (HIIT) and sprint interval training as protocols for improving VO_2_max in inactive subjects [10–12]. Moreover, Milanović et al. [13] found that high-intensity interval training is beneficial for VO_2_max in recreational subjects with lower baseline levels of VO_2_max (up to 40 mL·kg^-1^·min^-1^) as well as high levels (above 48 mL·kg^-1^·min^-1^), while moderate-intensity exercise is more suitable for moderately trained subjects (40–47 mL·kg^-1^·min^-1^). Thus, different training status may impact the efficiency of different training modes.

Nader [14] has reported that strength and endurance training produce diverse adaptations, with little overlap between them. Furthermore, when strength and endurance training are performed simultaneously, a potential interference in strength development may occur [14]. The training effects on body composition may also differ between different types of training, although increases in lean mass and decreases in fat mass, percent body fat and visceral fat has been observed for various types of training [8, 9, 15, 16]. HIIT and RUN training produce large energy expenditure [17], which results in overall decreases in body mass due to fat burning with minimal increases in muscle mass [18, 19]. On the other hand, strength training results in large increases in fat-free mass with relatively small effects on fat mass, at least after short-term training interventions [20]. However, large variability exists in response to different training protocols, due in the large part to genetics [21], gender differences [22] and the relationship between the magnitude of fat loss and the baseline body fat values [15].

Conventional training protocols affect a few selected heath profile components, usually either cardiovascular, metabolic or musculoskeletal fitness [21]. Thus, it is important to describe a training protocol that could increase a broad health profile spectrum. This type of training may be suggested as complex physical activity consisting of different movement patterns that stimulate both aerobic and strength performance. Also, performance related training modalities, such as recreational soccer, is a highly motivating and social activity which produce larger improvements of fitness compared to conventional training protocols [23]. Therefore, recreational soccer, as performance related activity, is ideal for addressing lack of motivation, a key component in physical (in)activity and immature levels of social habits due its popularity (~500 million players worldwide). Milanović et al. [23] concluded that recreational soccer could be a promising type of physical activity for overcoming barriers such as cost-efficiency, time-efficiency, access to facilities and intrinsic motivation.

Recreational soccer is a type of complex training that produces a positive physiological response in healthy subjects, patients and elderly people regardless of age, gender or training experience [24]. Despite the fact that recreational soccer has a large aerobic component with average heart rate ~80% HRmax, 20% of total training time comprises varied high-intensity movement patterns (e.g. high-intensity runs, stop-and-go actions, jumps, sprints, turns and other sport-specific actions) with average heart rate above 90% HRmax [25]. Krustrup et al. [26] reported that recreational soccer and endurance running produce similar increases in VO_2_max during the initial phase of training (first 4 weeks), namely 7% and 6%, respectively. However, a further increase (6%) was observed only in the recreational soccer group during the next 8 weeks, while the stimulus of factors determining VO_2_max during the running training was not large enough for additional increases [27, 28].

Previous studies provide valid evidence that playing soccer is effective for cardiorespiratory fitness, muscular adaptations [20, 29, 30], physical capacity and health profile [31] in various age categories and populations. Based on what is known about the potential benefits of recreational soccer, Krustrup et al. [25] published a topical review describing the effects of regular recreational soccer training on cardiorespiratory fitness, metabolic fitness and musculoskeletal fitness, and found positive effects on the given fitness components. However, this topical review comprises results from many randomized controlled studies studies performed with participants of varied age, gender, fitness status and health profile. Each of the studies used extensive testing procedures related to cardiovascular, metabolic and musculoskeletal fitness and health effects, but none of them investigated the effects of recreational soccer on all health-related physical fitness components as defined by the ACSM guidelines [1]. Accordingly, none of the studies provided a direct comparison of the full range of fitness effects of recreational soccer compared to other conventional training programmes, such as moderate-intensity continuous running. The purpose of this study was therefore to determine the effects of recreational soccer compared to RUN on maximal aerobic power, body composition, flexibility and jump performance in healthy untrained men. We hypothesised that the movement patterns and intermittent activities of recreational soccer could produce adequate stimuli to cause a broader fitness response than moderate-intensity continuous running.

## Methods

### Participants

**Fig 1:**
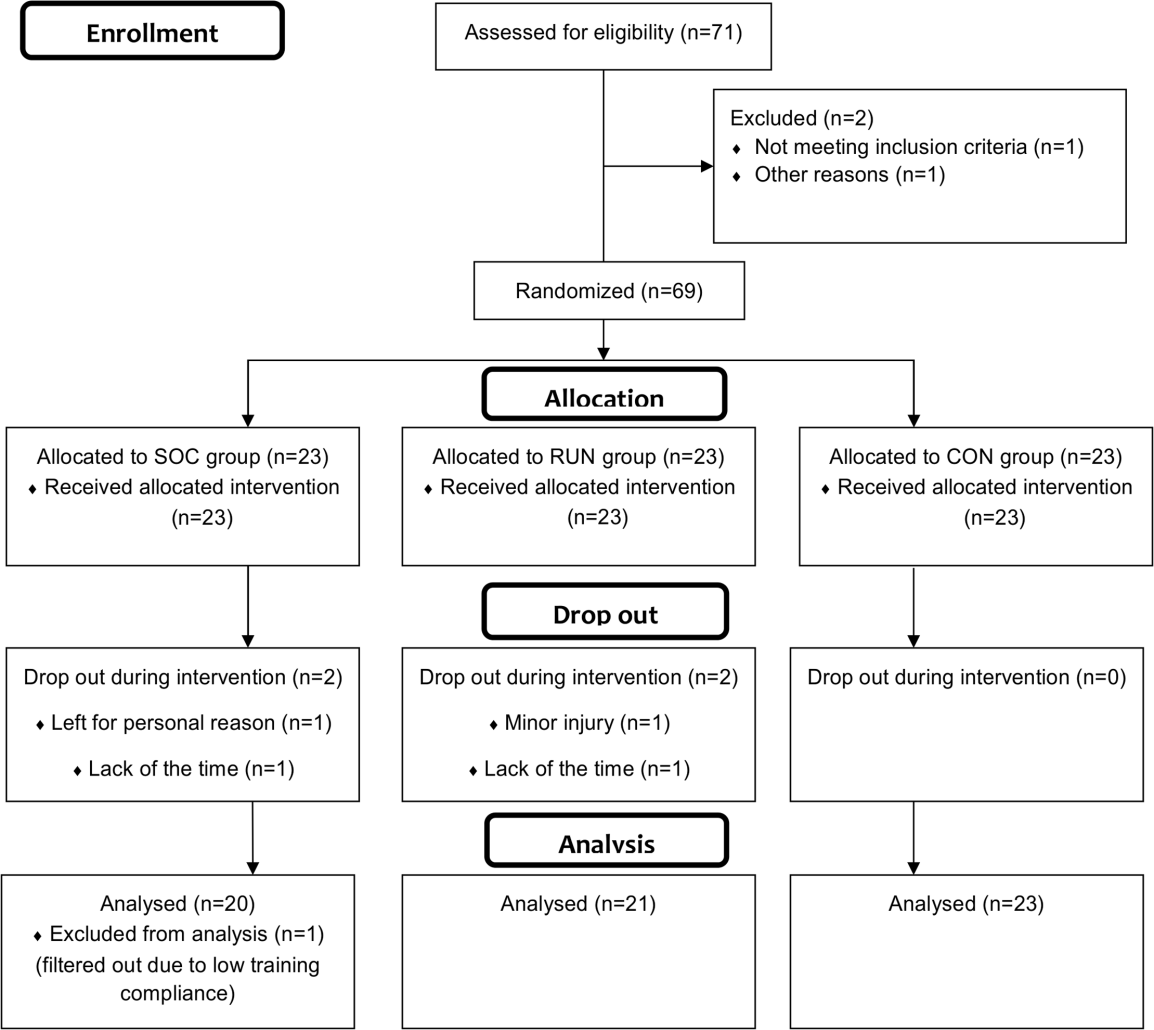
Flow chart diagram of participants’ enrolment, randomization and final analysis.

Sixty-nine healthy untrained males enrolled in this randomised controlled trial. Healthy, untrained, recreational, inactive and non-athletic men aged between 20 and 40 who were not suffering from any kind of acute or chronic diseases and had not participated in any type of regular physical training for at least 2 years were included. Exclusion criteria were: semi-active participants or semi-professional soccer players with any kind of training activity related to soccer; older than 40 years or younger than 20 years; any kind of injury within 3 months of the start of the training programme; female. All data related to inclusion and exclusion criteria were collected by a purposely designed questionnaire. Sixty-four participants completed the study after being randomly assigned to one of three groups: a soccer training group (SOC; n = 20); a running group (RUN; n = 21); or a control group performing no physical training (CON; n = 23). The process of enrollment, allocation, and drop-out as well as exclusion from analysis for any reason are shown in Fig 1. All subjects trained more than the minimum requirement of 50% of the training sessions for being included in the data treatment. For the participants who completed the study, no group differences were present at baseline. General descriptive parameters are presented in Table 1. The experimental protocol and associated risks were explained both orally and in writing to all subjects before they provided written consent. The study was approved by the Ethics Committee of the Faculty of Kinesiology at the University of Zagreb according to the Helsinki Declaration.

**Table 1:** General descriptive parameters.

	Soccer group (*n* = 20)		Running group (*n* = 21)		Control group (*n* = 23)	
	Initial	Final	Initial	Final	Initial	Final
Age (years)	34±4	34±4	32±4	32±4	30±3	30±3
Body height (cm)	178.6±4.2	178.6±4.2	179.4±7.0	179.4±7.0	177.8±8.0	177.8±8.0
Body mass (kg)	78.1±8.3	72.2±8.3	78.0±5.5	72.3±5.5	76.6±12.0	79.2±12.6

### Procedure

All participants, regardless of group assignment, were tested for each of the following physical fitness components: body weight, body mass index, fat-free mass, maximal aerobic power, minute ventilation, maximal heart rate, squat jump, countermovement jump with arm swing, sit-and-reach. All pre- and post-training testing procedures were completed in the same order, spaced 12 weeks apart. During testing, the air temperature ranged from 24°C to 27°C.

#### Cardiorespiratory fitness

Maximal oxygen uptake and ventilation were determined by a breath-by-breath pulmonary gas exchange system (Quark b^2^, COSMED, Italy) during an incremental treadmill test. HR was determined by the Polar Team System (Polar Electro Oy, Kempele, Finland). The starting speed was 3 km•h^-1^, with speed increments of 1 km•h^-1^ every 60 seconds. The subjects walked the first five steps (up to 7 km•h^-1^) and continued running from 8 km•h^-1^ until volitional exhaustion. During recovery after each test protocol, the subjects walked at 5 km•h^-1^ for 5 minutes. The last half or full stage that the subject could sustain (for either 30 or 60 s) was defined as the subject's maximal speed. Cardiorespiratory data ‒ maximal aerobic power, minute ventilation, maximal heart rate ‒ were filtered and averaged on a 5-second basis.

#### Muscular fitness

Muscular power was assessed by 2 jumping tests on a Kistler force platform (Kistler, Winterthur, Switzerland). The first test protocol was a squat jump (SJ) consisting of jumping from a fixed semi-squat position with the hands held at the hips. The second vertical jump test was a free countermovement jump with arm swing (CMJ) during which the participants were free to use their hands while jumping. Both tests were performed three times (three trials). The intra-class correlation coefficients for test-retest reliability for the SJ and CMJ tests were 0.93 and 0.95, respectively. A close correlation has been observed between jump performance and muscle strength for young men [32].

#### Flexibility

The sit-and-reach test was conducted indoors using a static sit-and-reach box supplied with a tape measure. The participant was instructed to sit with legs together and extended in front of him so that the feet (shoes off) touched the first step. Both knees were held together and flat on the floor. The scale (in centimetres) for measuring the distance was drawn on the first step. The end of the feet, i.e. the beginning of the step, representing the starting point of the scale, was regarded as point zero. All centimetres above zero were positive, whereas those below, towards the knees, were negative. The task was to perform the furthest possible front bend with arms extended and hands on top of each other, palms facing downward. That position was held for 2 seconds in order to measure the distance. The test was performed three times (three trials). The maximal reach distance was recorded in centimetres for all three trials. The purpose of this test was to assess the flexibility of the lower back and hamstring [33]. The intra-class correlation coefficient for test-retest reliability for the sit-and-reach test was 0.93.

#### Body composition

Anthropometric variables were measured according to the instructions of the International Biological Program (IBP) at the beginning of the study. Body height was measured to the nearest 0.1 cm. Body composition parameters were assessed by Tanita Body Composition Analyser (BC-418; Tanita, Tokyo, Japan). The Tanita BC-418 body fat analyser measures impedance across both arms, legs and trunk via multiple frequencies of 50 kHz. The system’s eight electrodes are in the form of footpads, and each footpad is divided in half so that the anterior and posterior portions form two separate electrodes. Impedance and body mass are automatically measured, and the subject’s height and age are manually entered into the system. Percent body fat measured using the Tanita BC-418 has been shown to correlate highly with the reference measure of dual-energy X-ray absorptiometry [34]. The participants were asked to observe the following before body composition measurement, as described by Rech et al. [35]: not to perform any physical exercises for 12 hours before testing; not to eat or drink anything for 4 hours before the evaluation; to urinate within 30 minutes of the evaluation; not to take any diuretics during the 7 days prior to testing; and not to consume alcohol for 48 hours before testing.

### Training Programme

Outdoor training was performed three times per week over 12 weeks. The participants in the intervention groups performed a 12-week training programme, whereas the participants in CON continued normal everyday activities during the study period. All training sessions were supervised by trained exercise instructors who finished bachelor or master study of kinesiology. All instructors were familiar with both recreational soccer and running training methods and well educated to delivery practical lessons. One instructor per match was delegated to supervise training, perform duties of referee and careful records each participant’s workout performance.

Soccer sessions consisted of 60 minutes of ordinary five-a-side, six-a-side or seven-a-side matches on a 20–45 m wide and 45–60 m long plastic grass pitch, with adjustments of the pitch size to 80–100 m^2^ per players [36, 37]. All games were played without consideration for the player’s playing position. There were no modifications to the general rules of soccer. Each training session began with a 10-minute low-intensity warm-up period, following which the participants completed four playing periods each lasting 10 minutes separated by 2-minute recovery periods. The warm-up consisted of 5 minutes of light running and passing the ball between players, 3 minutes of dynamic and static stretching and another 2 minutes with 2–4 acceleration runs. The heart rate of the participants was measured continuously during all training sessions using heart rate belts (Polar Team System, Polar Electro Oy, Kempele, Finland). The average heart rate during recreational soccer training was 81±4% of the individual maximal heart rate, equivalent to 151±6 bpm. Around 20% of the total training activity during recreational soccer was carried out at an intensity above 90% HRmax.

Running sessions consisted of 60 minutes of continuous moderate-intensity running in the park, with an average heart rate similar to that of SOC (~80% HRmax) (measured with Polar Electro, Kempele, Finland). Each training session began with a 10-minute low-intensity (~65% HRmax) warm-up period comprising walking and jogging. The total number of training sessions did not differ between groups and was 31.3±2.2 and 29.8±3.1 (out of a possible 36 total sessions) for participants in SOC and RUN, respectively. The training programme was designed to conform in principle to that recommended by the American College of Sports Medicine [38]. A more detailed description of the training programme is presented in Table 2. There was no difference in the training volume, intensity and frequency, which represents an important factor when comparing the effects of these two groups.

**Table 2:** Description of the training programmes for soccer and running group.

	Soccer group	Running group
**Frequency**	3 times / week	3 times / week
**Duration**	60 min (10 min warm up 4x10 min exercise, rest 2 min)	60 min (10 min warm up; 40 min running; 10 min cool down)
**Intensity**	~ 80 (65–100) % HRmax	~ 80 (65–85) % HRmax
**Type of activity**	Five-a-side, six-a-side or seven-a-side matches on a 30–45 m wide and 45–60 m long pitch	Continuous moderate intensity running

### Statistical Analysis

Data analysis was performed using the Statistical Package for the Social Sciences (v13.0, SPSS Inc., Chicago, IL, USA). Descriptive statistics, Kolmogorov–Smirnov (normality of the distribution) and Levene’s (homogeneity of variance) tests were calculated for all experimental data before inferential testing. Changes in health-related physical fitness parameters were compared over the training period for players in the two experimental and control groups using two-factor (group x time) univariate analysis of variance (ANOVA). If the appropriate statistical significance was identified, then the Bonferroni post-hoc test was used to further distinguish the differences among groups. Cohen *d* effect sizes (ES) were also calculated to determine the magnitude of the group differences in health-related physical fitness. ES was classified as follows: <0.2 was defined as trivial; 0.2–0.6 was defined as small; 0.6–1.2 was defined as moderate; 1.2–2.0 was defined as large; >2.0 was defined as very large; and >4.0 was defined as extremely large [39]. The Kolmogorov-Smirnov tests showed that data were normally distributed, and no violation of homogeneity of variance was found using Levene’s test. The statistical significance was set at p<0.05.

## Results

No differences between groups for all health-related physical fitness components were observed at baseline (Table 3). Average heart rate was ~80% HRmax in SOC as well as RUN. In SOC, heart rates were above 90%HRmax for 20% of total training time, whereas heart rate was not above 90%HRmax during in RUN.

**Table 3:** Health-related physical fitness components for soccer, running and control group before and 12-week training intervention.

	Soccer group (*n* = 20)				Running group (*n* = 21)				Control group (*n* = 23)			
	Initial	Final	Δ (%)	ES	Initial	Final	Δ (%)	ES	Initial	Final	Δ (%)	ES
Body mass (kg)	78.1±8.3	72.2±8.3^a^	-7.5%	-0.70	78.0±5.5	72.3±5.5^b^	-7.4%	-1.05	76.6±12.0	79.2±12.6	3.5%	0.22
Body mass index (kg·m^-2^)	24.45±2.20	22.60±2.20^a^	-7.5%	-0.84	24.29±1.93	22.49±1.85^b^	-7.4%	-0.96	24.12±2.89	24.96±3.07	3.5%	0.28
Fat free mass (%)	76.23±1.35	80.90±3.44^a^	6.1%	1.79	75.54±1.72	79.93±3.34^b^	5.8%	1.66	76.56±3.66	78.11±4.20	2.0%	0.39
VO_2_max (L·min^-1^)	3.00±0.67	3.42±0.68^a^	13.9%	0.62	3.16±0.55	3.53±0.48^b^	11.8%	0.72	2.96±0.63	2.85±0.60	-3.7%	-0.18
Aerobic power (mL·kg^-1^·min^-1^)	38.15±6.23	47.40±8.98^a^	24.2%	1.20	40.50±6.63	49.22±8.19^b^	21.5%	1.17	38.65±4.74	36.85±9.51	-5.0%	-0.24
Maximal heart rate (b.p.m.)	189.2±8.3	193.0±8.3	2.0%	0.46	190.4±5.6	185.1±5.6	-2.8%	-0.94	189.5±7.3	186.6±7.4	-2.0%	-0.39
Minute ventilation (L)	137.1±17.5	155.7±22.0^a^	13.5%	0.94	142.45±9.53^b^	155.70±12.49	9.3%	1.19	137.4±16.6	137.7±16.6	-1.0%	0.02
Squat jump (cm)	39.66±5.00	45.54±5.83^a^ ^,^ ^c^	14.8%	1.08	39.75±4.90	41.08±6.46	3.3%	0.23	40.20±3.67	40.30±4.82	0.3%	0.02
CMJ with arm swing (cm)	44.40±6.07	49.78±7.15^a^ ^,^ ^c^	12.1%	0.81	44.47±5.92	45.79±7.54	3.0%	0.19	45.15±6.22	45.24±6.55	0.2%	0.02
Sit and reach (cm)	6.68±6.37	12.95±6.57^a^ ^,^ ^c^	94.0%	0.97	6.97±3.90	7.10±3.82	1.8%	0.03	6.28±4.16	6.30±4.37	0.3%	0.02

VO_2_max–maximal aerobic power; CMJ–countermovement jump; Δ (%)–percent changes between initial and final measurement; ES–effect size

^a^–significant difference between soccer and control group (p<0.05)

^b^—significant difference between running and control group (p<0.05)

^c^—significant difference between soccer and running group (p<0.05)

**Fig 2:**
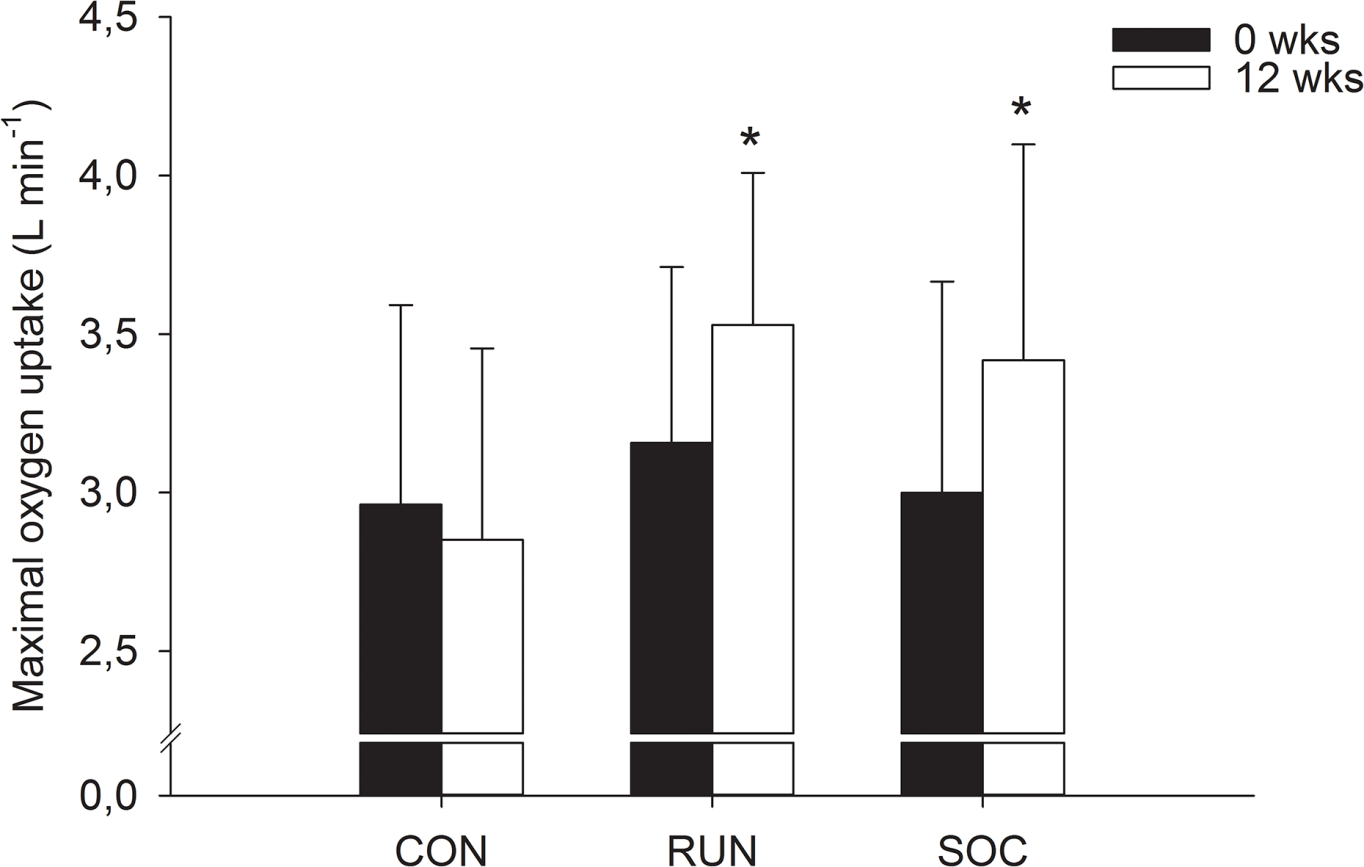
Differences among soccer (SOC), running (RUN) and control (CON) group in VO_2_max absolute values (L·min^-1^); *—denotes significant difference compared to baseline (p<0.05).

Over the 12 weeks, VO_2_max relative to body weight increased more (p<0.05) in SOC (24.2%, ES = 1.20) and RUN (21.5%, ES = 1.17) than in CON (-5.0%, ES = -0.24), partly due to large changes in body mass (-5.9, -5.7 and +2.6 kg, p<0.05, for SOC, RUN and CON, respectively) (Table 3). The increases in absolute values of maximal aerobic power were 418±65 and 373±75 mL**·**min^-1^ in SOC and RUN, respectively, corresponding to 13.9 (ES = 0.62) and 11.8% (ES = 0.72), which was more than in CON (-111 mL**·** min^-1^ or -3.7%, ES = -0.18) (Fig 2). After 12 weeks, peak ventilation increased more (p<0.05) in SOC (13.5%, ES = 0.94) and RUN (9.3%, ES = 1.19) than in CON (-1.0%, ES = 0.02) (Table 3). The estimated increases in fat-free mass over the 12 weeks were 6.1% (ES = 1.79) and 5.8% (ES = 1.66), respectively, for SOC and RUN, higher than in CON (2.0%, NS, ES = 0.39) (Table 3).

**Fig 3:**
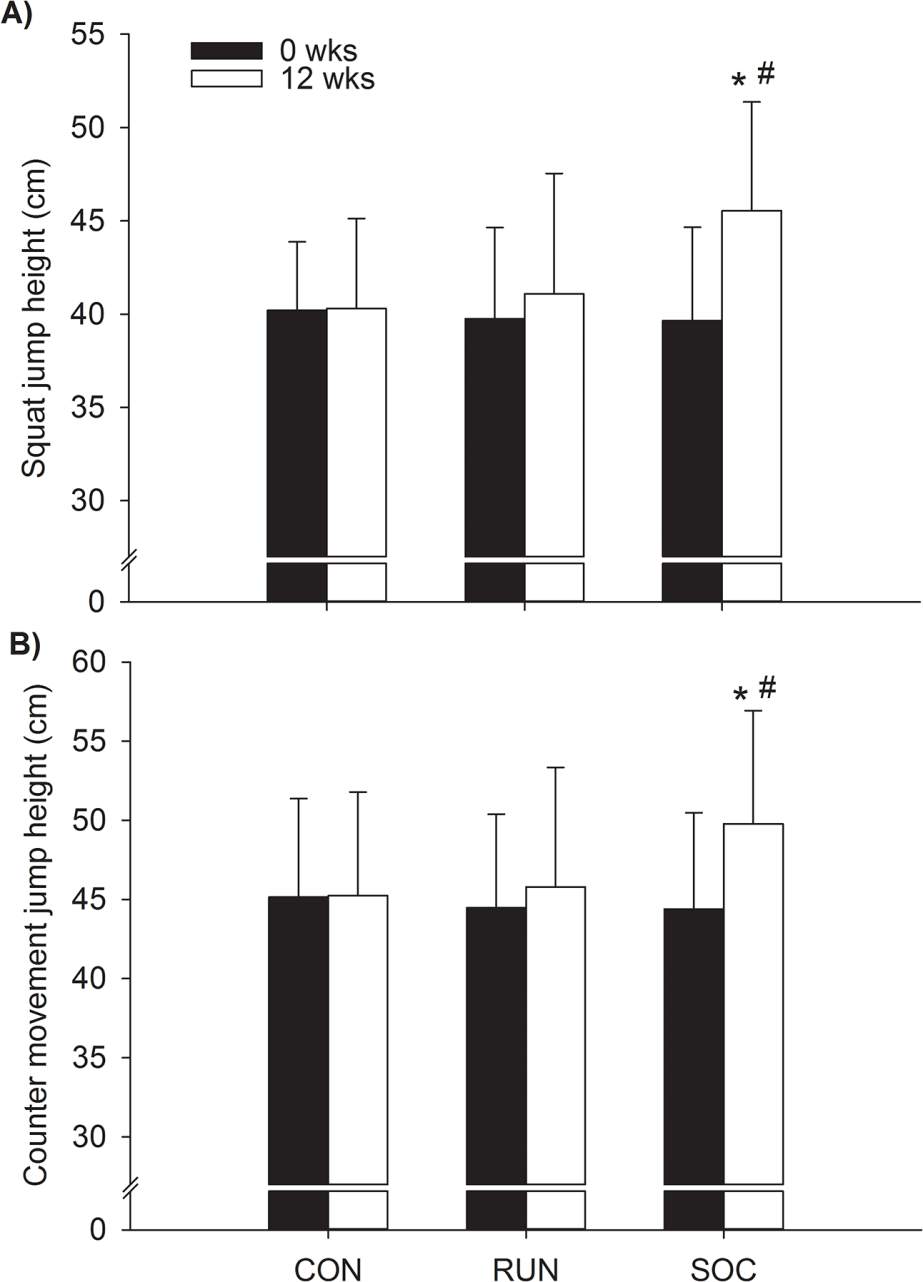
Differences among soccer (SOC), running (RUN) and control (CON) groups in: A) squat jump performance and B) countermovement jump with arm swing; *—denotes significant difference compared to baseline (p<0.05); #—significant difference between SOC and RUN group in post intervention (p<0.05).

**Fig 4:**
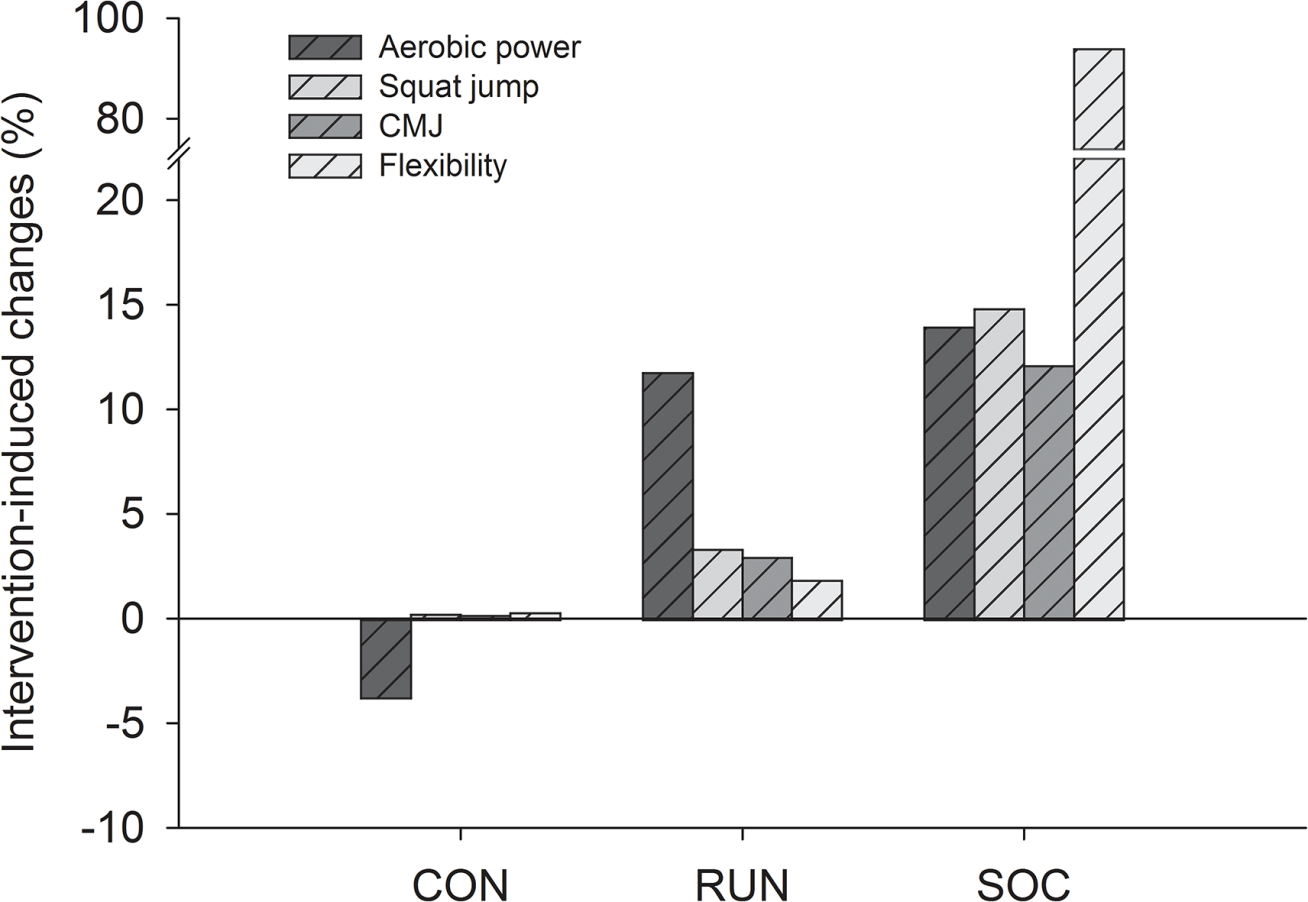
Intervention-induced changes in health related fitness parameters for soccer (SOC), running (RUN) and control (CON) group.

Over the 12 weeks, SJ performance increased more (p<0.05) in SOC (14.8%, ES = 1.08) than in RUN (3.3%, ES = 0.23) and CON (0.3%, ES = 0.02) (Fig 3). Likewise, CMJ-arm increased more (p<0.05) in SOC (12.1%, ES = 0.81) than in RUN (3.0%, ES = 0.19) and CON (0.2%, ES = 0.02) (Fig 3). Flexibility increased in SOC over the 12 weeks (94%, ES = 0.97), more than in RUN and CON (0–2%, ES = 0.02) (Fig 4).

## Discussion

The present study examined the effects of 12 weeks of recreational soccer compared to moderate-intensity continuous running on health-related physical fitness components in healthy untrained men. The major findings were that both training regimes caused large improvements in maximal aerobic power and a reduction in body mass. However, jump performance and flexibility were improved markedly more after the soccer training intervention in comparison to running.

Increases of 24% and 22% were observed in maximal aerobic power relative to body mass over the 12 weeks of soccer training and moderate intensity running, which is higher than the observed increases of 3–8% in other training studies with untrained young men applying moderate-intensity running [20] and circuit training [5] and also somewhat higher than the observed increases of 13–15% in studies with recreational soccer training [31] and high-intensity interval running [40]. This extraordinarily large training-induced increase in maximal aerobic power relative to body mass is explained by a combination of a large increase in maximal aerobic power and a marked decrease in body mass. The observed increase in absolute values for maximal aerobic power after soccer training was 418 mL min^-1^, corresponding to 14%,which is similar to previous studies involving 12–16 weeks of soccer training [25, 31], whereas the decline in body weight of 5–6 kg is larger than previously observed in untrained men (1–2.5 kg) [31, 40]. Thus, the present study confirms previous findings that recreational soccer training has a high cardiovascular loading and is an efficient aerobic training protocol [41]. Aerobic training has also been shown to increase fat oxidation after both types of training applied in the present study [31, 40]. In the present study, the training volume was somewhat higher than in previous studies due to a high training attendance of nearly three training sessions per week, which may partly explain the somewhat higher increase in absolute VO_2_max values of 373 mL min^-1^, corresponding to 12%, as well as the large body weight decrease. However, the latter difference may also be associated with dietary alterations during the intervention period, which cannot be ruled out since diet was not controlled in the present study.

In the present study, the average heart rate response during training was ~80%, with no difference in average cardiovascular loading between SOC and RUN. However, the soccer training induced a heart rate loading >90% HR_max_ for more than 20% of the total training time, which was higher than in RUN and in accordance with previous findings after soccer training [25, 31]. Despite these differences between the intervention groups, there was a similar increase in VO_2max_, indicating that continuous moderate-intensity exercise can produce the same improvements in maximal aerobic power as aerobic high-intensity training when the average cardiovascular loading is the same, at least for participants with low-to-moderate baseline values for VO_2max_. In the present study, the peak ventilation was improved by 19 and 13 L min^-1^ in the soccer and moderate-intensity running groups, which is very similar to the improvements of 17 and 15 L min^-1^ observed in a previous study [20].

An interesting finding in the present study was the large increase in squat jump and countermovement jump performance of 12–14% in SOC, which was much greater than in the running and control groups, where there were no changes in jump performance. Thus, in addition to the marked changes in aerobic power and body mass, the soccer training resulted in positive adaptations in explosive muscle power. In other training studies, there is clear evidence of an elevated muscle mass after a period of soccer training [31, 42], but discrepancies in relation to improved jump performance [31, 42]. For example, some studies have shown that jump performance does not improve during the first 12–16 weeks of recreational soccer training in men and women [31, 42], but does improve moderately after longer-term training [41, 43]. In contrast, improvements have been observed after 14 weeks of soccer training in inactive women [44]. Jump performance increased 12–14% in the present study, which may be partly related to the decrease in body mass. However, the increase in jump performance was larger than the 6% decrease in body mass, and the improvement in jump performance was much greater in the soccer group than in the running group despite similar changes in body mass and body composition in the two groups. Small-sided recreational soccer training has been shown to include few jumps, but multiple sprints, turns, tackles, shots, accelerations and decelerations [41, 45], which may all improve explosive strength. Actually, about 200 of these specific intense actions have been reported per hour of recreational small-sided soccer training [41, 45]. Thus, recreational soccer training appears to involve some of the same components as complex training regimes that have been shown to improve rate of force development, speed and agility. Moreover, glycogen depletion pattern analyses after recreational and sub-elite soccer training have shown a high involvement of the type 2 fibres [41, 46]. Thus, a higher loading of the type 2 fibres in the soccer training group may explain the greater adaptive response in comparison to the running group.

The notion that recreational soccer training provides broad-spectrum fitness effects was further supported by the finding that flexibility was improved after 12 weeks of training. The magnitude of the improvement corresponded to training responses after stretching protocols [47]. Few studies have investigated the effects of recreational soccer training on flexibility, and one recent study involving middle-aged female hospital employees showed no effect on flexibility after 12 weeks of soccer training [48]. In the soccer group, jump performance was higher after the intervention, with concomitant improvements in flexibility, so part of the increased ability to perform muscle work requiring a high rate of force development may be explained be an increased flexibility, as suggested by others [49].

Despite many benefits observed in this study there are few limitations. Firstly, we have investigated only male participants aged between 20–40 yrs while female adults were not included. Secondly, only health-related physical fitness components were assessed. Thirdly, we compared recreational soccer only with continuous moderate-intensity running despite that for some fitness components other conventional training programmes are much better. Future studies should consider effects of recreational soccer compared to other conventional training programmes such as HIIT, strength training as well as concurrent training. Also, further research are required to investigate whether recreational soccer is suitable for long term maintenance of physical fitness components and whether is it possible to reduce the number of training session without marked decreases of performance elicited by previous recreational soccer interventions. Finally, effects of recreational soccer on skill related physical fitness components are still unclear.

In conclusion, the present study showed a very large improvement in maximal aerobic power, a decrease in body mass and an increase in fat-free mass after 12-week interventions involving soccer training and moderate-intensity running in untrained men. Moreover, soccer training improved jump performance and flexibility markedly and to a greater degree than running. Together, these findings provide clear evidence that soccer is an intense multi-component type of training with broad-spectrum fitness benefits for untrained men.
